# Identification of a novel ESR1 mutation in a Chinese PCOS woman with estrogen insensitivity in IVF treatment

**DOI:** 10.1186/s12958-022-01029-7

**Published:** 2022-11-18

**Authors:** Faying Liu, Lifeng Tian, Jun Tan, Zengming Li, Haiyan Qin, Dingfei Xu, Zhihui Huang, Xingwu Wu, Ge Chen, Qiongfang Wu, Yang Zou

**Affiliations:** 1 Key Laboratory of Women’s Reproductive Health of Jiangxi Province, Jiangxi Maternal and Child Health Hospital, Nanchang, China; 2 Central Laboratory, Jiangxi Maternal and Child Health Hospital, Nanchang, China; 3 Reproductive Medicine Center, Jiangxi Maternal and Child Health Hospital, Nanchang, China; 4 Health Examination Center, Jiangxi Maternal and Child Health Hospital, Nanchang, 330006 Jiangxi China

**Keywords:** Polycystic ovary syndrome, Whole exome sequencing, *ESR1* mutation, Estrogen insensitivity

## Abstract

**Background:**

Polycystic ovary syndrome (PCOS) is a complex reproductive disorder, that affects approximately 5–10% of women of reproductive age. The disease is complex because its evolution may be impacted by genetic, lifestyle and environmental factors. Previous studies have emphasized the important roles of estrogen receptors in the pathogenesis of PCOS.

**Objective:**

To use whole exome sequencing (WES) to assess possible pathogenic factors in a PCOS patient who exhibited estrogen insensitivity during hormone replacement therapy (HRT) treatment.

**Methods:**

Genome sequencing and variant filtering via WES were performed in a patient with PCOS. DNA extraction from 364 unrelated female controls without PCOS was followed by PCR amplification, Sanger sequencing and sequence alignment. Evolutionary conservation analysis, protein structural modelling and in silico prediction were applied to analyse the potential pathogenicity of the novel *ESR1* mutation.

**Result(s):**

During the controlled ovarian hyperstimulation (COH) period of an IVF cycle, the patient experienced markedly prolonged ovarian stimulation due to a poor response to gonadotropins (Gn) and elevated serum FSH. A novel heterozygous *ESR1* mutation, c.619G > A/p.A207T, leading to the replacement of a highly conserved alanine with a threonine, was identified in this patient, via WES analysis. This novel variant was not identified in 364 unrelated female controls without PCOS, or in the Exome Aggregation Consortium (ExAC) or 1000 Genome Project.

**Conclusion(s):**

We identified a novel heterozygous *ESR1* mutation in a Han Chinese PCOS woman exhibiting clinical signs of estrogen insensitivity. This study may provide new strategies for IVF therapy, especially for patients who exhibit estrogen insensitivity during IVF cycle.

## Introduction

Polycystic ovary syndrome (PCOS) is a complex reproductive disorder, that affects approximately 5–10% of women of reproductive age [1]. It is characterized by hyperandrogenemia, chronic anovulation, oligomenorrhea/amenorrhea, and multiple cysts in the ovaries as observed via ultrasound examination and thus is one of the major causes of female subfertility [2]. The disease is complex as genetic, lifestyle and environmental factors all contribute to its aetiology [3–5].

Estrogen regulates diverse physiological functions in vertebrates, especially in the reproductive system [6]. The physiological functions of estrogen are mediated mainly by estrogen receptor alpha (ERα, ESR1) and estrogen receptor beta (ERβ, ESR2), both of which thought to be ligand inducible transcription factors that can dimerize and regulate the transcription of multiple downstream target genes [7, 8]. Previous studies showed that ERα knockout led to the development of PCOS in female mice, with multiple cystic follicles in the ovary and elevated levels of luteinizing hormone (LH) [9], while ERβ disturbance caused partially arrested follicular development and compromised fertility in female mice [10]. In addition, other studies have suggested that the expression of ERα was lower in proliferative endometria of PCOS patients than in control women [11], and that ERα and ERβ expression was significantly lower in PCOS granulosa cells than control granulosa cells [12]. These studies emphasized the important roles of the estrogen receptors in the pathogenesis of PCOS.

To date, germline mutations in the *ESR1* and *ESR2* genes have been reported in samples from only a few sporadic and hereditary PCOS cases [13–16]. A germline mutation in *ESR2* was identified in three members with hereditary medullary thyroid carcinoma and this mutation was shown to promote cell proliferation in MCF-7 cells [16]. While only 5 females [14, 15, 17] and 2 males [13, 15] were reported to harbor *ESR1* germline mutations, all these mutations were homozygous and associated with multicystic ovaries and delayed pubertal mammary gland development in females; furthermore, the *ESR1*-mutated women presented with complete estrogen insensitivity, and elevated levels of serum estrogen, follicle-stimulating hormone (FSH) and luteinizing hormone (LH) [14, 15, 17].

In the present study, we report a Han Chinese woman with PCOS exhibiting clinical signs of estrogen insensitivity in adjuvant therapy during the IVF cycle. Via whole exome sequencing (WES) analysis, we identified a novel heterozygous *ESR1* mutation, c.619G > A/p.A207T, leading to the replacement of a highly conserved alanine with threonine at the 207th residue in this patient.

## Materials and methods

### Patient and hormone tests

Peripheral blood was collected from the PCOS sample with estrogen insensitivity in the Reproductive Medicine Center, Jiangxi Provincial Maternal and Child Health Hospital (Nanchang, China). Additionally, 364 unrelated female controls without PCOS were recruited from Jiangxi Maternal and Child Health Hospital. All participants provided written informed consent. This study complies with the Declaration of Helsinki and was approved by the Institutional Review Board of Jiangxi Provincial Maternal and Child Health Hospital. Before the initiation of therapy, the basal serum levels of estradiol (E2), FSH, LH, thyroid stimulating hormone (TSH), progesterone (PRGO), prolactin (PRL), testosterone (T), thyrotropin (TSH), free triiodothyronine (FT3), free thyroxine (FT4) and cancer antigen 125 (CA125), were measured by commercial kits as described previously [18]. Anti-Mullerian hormone (AMH) was measured by an automated Roche Elecsys instrument (Roche; Elecsys).

### WES

Genomic DNA (gDNA) was isolated from peripheral blood leukocytes with a Blood DNA kit (OMEGA Bio-tek Inc., Doraville, GA, USA) according to the manufacturer’s instructions. The gDNA sample was quantified with a NanoDrop 2000 fluorospectrometer (Thermo Fisher Scientific, MA, USA). A total of 3 μg of DNA sample was used for high-throughput sequencing. Subsequent exome capture, library construction and high throughput sequencing were carried out at the Beijing Genomics Institute (BGI, Shenzhen, China). Prior to high-throughput sequencing with a BGISEQ-500 sequencer, exome capture was performed with a BGI Exome V4 Kit (59 Mb target region) and the sequencing libraries were constructed by the MGIEasy™ DNA Library Prep Kit V1 (BGI, Shenzhen, China, Cat No. 85–05533-00). The sample had an average coverage depth of ~ 100 x. The sequenced data were aligned to the human reference genome (GRCh37/hg19). The common variants (minor allele frequency > 0.01) found in the 1000 Genomes Project (1000G, http://www.1000genomes.org), dbSNP147 (https://www.ncbi.nlm.nih.gov/projects/SNP/) or ExAC (http://exac.broadinstitute.org/) were excluded for further analysis.

### Candidate gene selection

Three online bioinformatics programs, MutationTaster (www.mutationtaster.org) [19], SIFT (http://sift.jcvi.org/) [20] and PolyPhen-2 (genetics.bwh.harvard.edu/pph2) [21], were applied to predict the potential pathogenicity of the rare variants/mutations. These programs automatically predict whether rare variants/mutations are likely pathogenic or benign.

### Sanger sequencing

Sanger sequencing was used to confirm the presence of the variant. Meanwhile, gDNA was isolated from the peripheral blood samples of 364 unrelated female controls without PCOS with the Blood DNA kit (OMEGA Bio-Tek Inc., Doraville, GA, USA) according to the manufacturer’s instructions. A 302 bp PCR amplicon spanning exon 2 of the *ESR1* gene (NM_001291230.1) was amplified with a pair of primers (*ESR1*_Forward: 5′-ttctaatgttaatggatt − 3′/*ESR1*_Reverse: 5′-ttcctcagtcgctttggctc-3′). PCR was carried out in 30 μl reactions containing 50 ng gDNA as the template, 1 × PCR buffer, 0.5 μM of each forward and reverse primer, 2.5 mM of MgCl_2_ (Takara Biotechnology), 2.5 mM dNTPs (Takara Biotechnology), and 1 U LA Taq (Takara Biotechnology). The amplification program for the PCR was performed in a Thermal Cycler 2720 (Applied Biosystems; Thermo Fisher Scientific, Inc., Waltham, MA, USA) with a three-step PCR protocol: an initial denaturation phase at 94 °C for 3 min, followed by 35 cycles of denaturation at 94 °C for 30 sec, annealing at 53 °C for 30 sec and extension at 72 °C for 30 sec, and ending with an extension phase at 72 °C for 10 minutes. The PCR products were visualized on a 1.5% agarose gel stained with ethidium bromide. The PCR products were then purified with a DNA purification kit (Tiangen, Beijing, China) and sequenced in both directions on an ABI 3730 XL Automatic Capillary DNA Sequencer (Applied Biosystems; Thermo Fisher Scientific, Inc., Waltham, MA, USA) with a BigDye terminator v3.1 Cycle Sequencing kit (Applied Biosystems, Foster City, Calif, USA). The sequencing data were assembled and aligned to the corresponding genomic sequence (*ESR1*, NM_001291230.1) with the SeqMan II program in the LaserGene (DNAStar, Madison, WI, USA).

### Evolutionary conservation analysis

The ESR1 protein sequences from 18 different vertebrate species, including *Homo sapiens* (NP_000116), *Pan troglodytes* (XP_009450519), *Mus musculus* (NP_001289460), *Rattus norvegicus* (NP_036821), *Ovis aries* (NP_000116), *Bos taurus* (NP_001001443), *Gallus gallus* (NP_990514), *Sus scrofa* (NP_999385), *Canis lupus familiaris* (NP_001273887), *Equus caballus* (NP_001075241), *Tupaia chinensis* (NP_001304001), *Mustela putorius furo* (XP_004753629), *Oryctolagus cuniculus* (XP_008261925), *Pongo abelii* (XP_002817538), *Coturnix japonica* (NP_001310118), *Alligator sinensis* (XP_014375965), *Ceratotherium simum simum* (NP_001266182) and *Xenopus tropicalis* (NP_988866), were subjected to evolutionary conservation analysis. Multiple sequence alignment was carried out with Molecular Evolutionary Genetics Analysis (MEGA) software (version 7.0) developed by the laboratory of Dr. Kumar [22].

### Protein structural modelling

The PDB file of ESR1 was generated by SWISS-MODEL in the ExPASy database (http://www.expasy.org) based on the protein sequences of human ESR1. With the generated PDB file, the protein structure of ESR1 was generated by DeepView Swiss-PdbViewer 4.0 software, by selecting “show dots surface”, “show backbone oxygen” and “sender in solid 3D”. Within the structure of the wild-type ESR1 protein, mutated ESR1 protein (p.A207T) was generated by changing alanine to threonine at the 207th residue.

## Results

### Clinical findings

The patient was 36 years old and was diagnosed with PCOS when she was undergoing in vitro fertilization (IVF) treatment due to secondary infertility. Her height was 158 cm and weight was 53 kg, and her body mass index (BMI) was 21.23 (within the normal rang for adult women, 18.5–24.9). She gave birth to a daughter when she was 20 years old, and then suffered from three spontaneous abortions between 2013 and 2016. Before the initiation of therapy, the laboratory tests showed that she had a mildly elevated level of basic serum FSH (14.56 IU/L, normal follicular phase, 3.5 to 12.5) and AMH (6.45 ng/mL, normal follicular phase, 0.777 to 5.24), while the levels of other associated factors, including E_2_, LH, TSH, PRGO, PRL, T, TSH, FT3, FT4 and CA125, were within the normal range on the third day of menstruation (Table 1).

**Table 1:** Laboratory test values for the PCOS patient with ESR1 mutation

Variable	PCOS sample	Normal range
Height (cm)	158	–
Weight (kg)	53	–
BMI	21.23	18.5–24.9
FSH (IU/L)	14.56	3.5–12.5
Estradiol (pg/mL)	41.17	12.4–233
LH (mIU/mL)	6.81	2.4–12.6
TSH (mIU/L)	0.98	0.27–4.2
PRGO (ng/mL)	0.7	0.2–1.5
PRL (ng/mL)	7.33	4.79–28.3
T (ng/dL)	38.58	7–75
FT3 (pg/mL)	3.17	2–4.4
FT4 (ng/dL)	1.44	0.93–1.7
AMH (ng/mL)	6.45	0.777–5.24
CA125 (u/mL)	13.77	0–35

*PCOS* Polycystic ovary syndrome, *BMI* Body mass index, *FSH* Follicle-stimulating hormone, *LH* Luteinizing hormone, *TSH* Thyroid stimulating hormone, *PRGO* Progesterone, *PRL* Prolactin, *T* testosterone, *FT3* Free triiodothyronine, *FT4* Free thyroxine, *AMH* anti-Mullerian hormone, *CA125* Cancer antigen 125

**Fig. 1:**
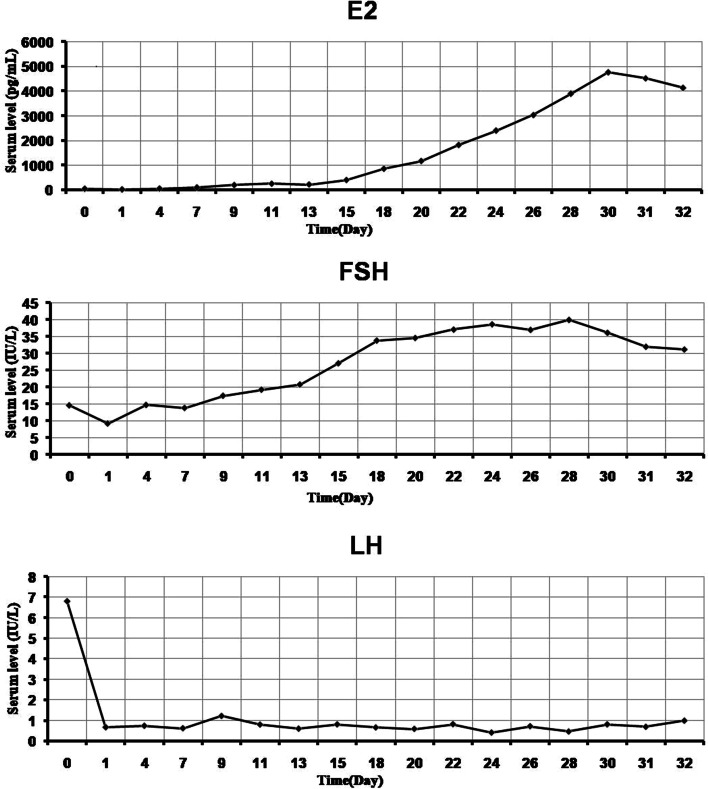
Fluctuating serum estrogen (E2), FSH and LH levels of the patient with PCOS during a controlled ovarian hyperstimulation (COH) regime in the IVF cycle. A prolong- acting agonist protocol was selected. Before the initiation of therapy, the baseline serum E2, FSH and LH levels were tested on the third day of menstruation (0). Twenty-eight days after pituitary downregulation with GnRHa (3.75 mg), the patient received 31-day Gn administration and exhibited slowly increasing and fluctuating levels of serum E2 in the cycle

A “long-term” GnRH-a regimen protocol was selected, Pituitary downregulation with GnRH-a (3.75 mg, long acting Diphereline, Beaufour Ipsen, France) on Day 3 of the menstrual cycle, and 28 days later, endocrine examination showed that the serum FSH of the patient was still high (9.18 IU/L, reference value < 5 IU/L). The patient was injected with recombinant human FSH (r-FSH, 112.5 IU/d, Gonal-F, Merck Serono, Switzerland) for 6 days, and the follicles did not grow significantly. Then, human menopausal gonadotropin (HMG, 75 IU/d, H20045720, China) was injected for 4 days, but the follicles continued to develop slowly. The dose of HMG was increased (400 IU/d) and treatment continued for 24 days, only one dominant follicle (follicular diameter > 1.8 cm) developed, while other follicles were less than 1.4 cm in diameter. Afterwards, Gn was adjusted to HMG (375 IU/d) and recombinant human lutropin (r-LH, 75 IU/d, Luveris, Merck Serono, Switzerland) due to the low level of serum LH (0.45 IU/L). At day 31 of ovulation induction, transvaginal ultrasound monitors showed that bilateral follicles of the patient grow poorly, while E2 levels continued to decline (from 4763 pg/mL to 4132 pg/mL). Generally, during the controlled ovarian hyperstimulation (COH) regime in the IVF cycle, the patient had a markedly prolonged ovarian stimulation period due to poor response to Gn and elevated serum FSH, who experienced a 31-day-Gn administration after pituitary down-regulation with GnRH-a and exhibited slowly increased and fluctuant serum level of estrogen in the cycle (Fig. 1). These clinical features suggest an estrogen insensitivity condition.

### Exome sequencing and sanger sequencing validation

**Fig. 2:**
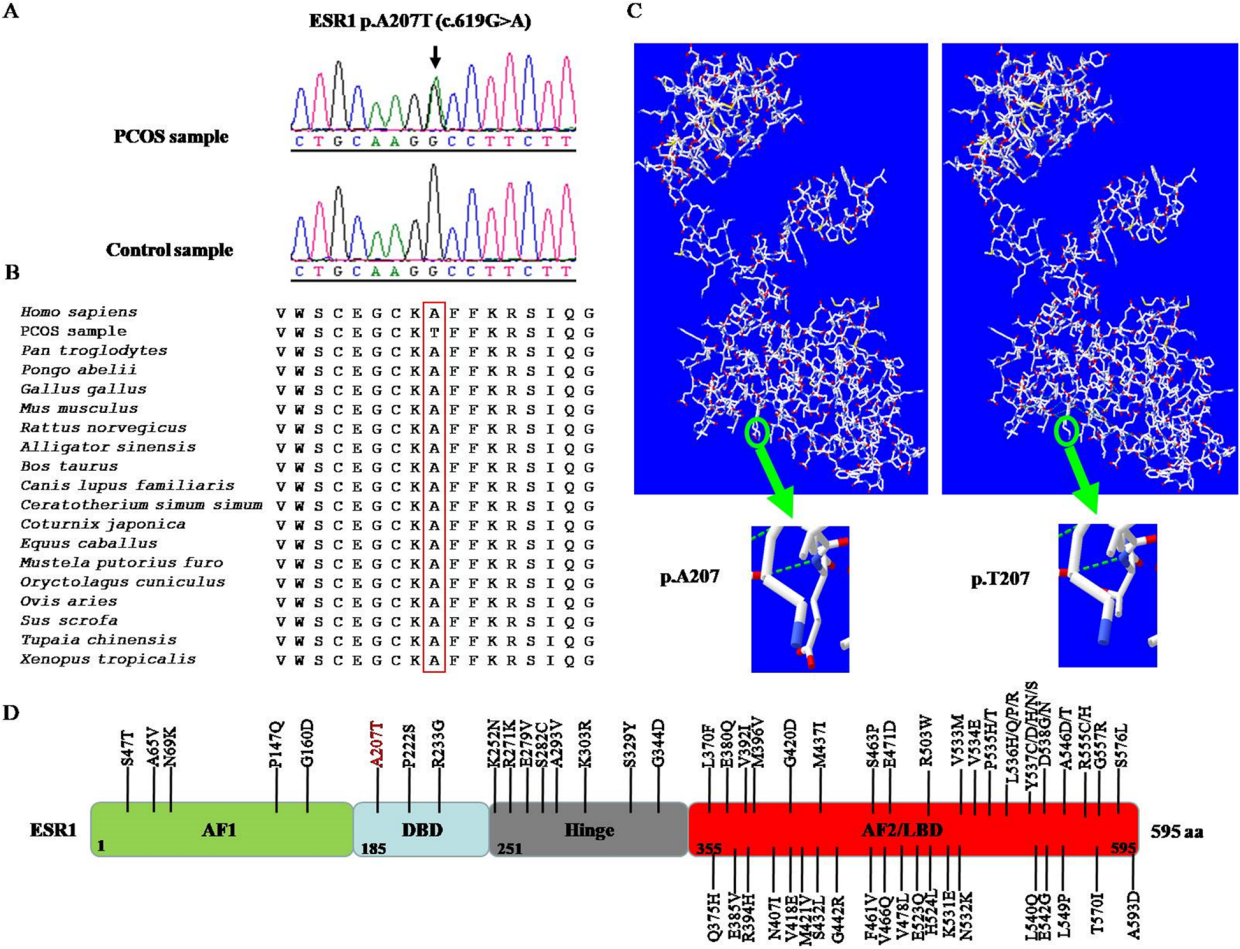
Evolutionary conservation analysis and protein structural modelling of the *ESR1* (p.A207T) mutatiom. **A** Representative sequencing electropherogram of the *ESR1* (p.A207T) mutation. The arrow indicates the location of the mutation. **B** Evolutionary conservation analysis of the *ESR1* (p.A207T) mutation. Sequence alignment with other species indicates that the affected Ala (A) 207 residue is highly conserved in all 18 vertebrate species. **C** Structural difference between wild type ESR1 (p.A207) and mutated ESR1 (p.T207) proteins. The protein structures of wild type and mutated ESR1 proteins were modelled based on the crystal model of the human ESR1 protein. **D** Location of ESR1 missense mutations found in clinical samples. The A207 residue (red text) shows the novel mutation identified in our study. AF1, activation function 1; DBD, DNA binding domain; AF2, activation function 2; LBD, ligand binding domain

A total of 26.99 million raw reads were produced by sequencing, and over 96% of the sequenced bases possessed a quality score of Q20. The raw sequencing data generated more than 5000 Mb of effective data and the average sequencing depth on target areas was over 90×. In total, 1419 SNPs and 362 indels, including 1398 SNPs and 274 indels in the exonic regions, and 93 SNPs and 88 indels in splicing sites, were obtained. For the identified SNPs and indels, the synonymous variants and common variants (MAF > 0.01) deposited in the dbSNP147, 1000 Genomes Project and ExAC databases, were excluded. For the remaining nonsynonymous variants, we evaluated their potential pathogenicities with a combination of the SIFT, PolyPhen-2 and MutationTaster prediction programs. Based on these filtering criteria, a novel heterozygous missense variant in the exon 4 in the *ESR1* gene (NM_001291230.1), c.619G > A (p.A207T), was identified. The identified *ESR1* variant was validated by direct Sanger sequencing of the patient sample (Fig. 2A). This novel variant was not identified in 364 unrelated female controls without PCOS, or in the ExAC database and 1000 Genomes Project databases.

### Evolutionary conservation analysis and protein structural modelling

Evolutionary conservation analysis based on 18 vertebrate species, from *Homo sapiens* to *Xenopus tropicalis,* showed that the *ESR1* A207T mutation changed a highly conserved alanine to threonine at the 207th residue (Fig. 2B). The results of protein structural prediction showed that the *ESR1* A207T mutation caused marked structural change when compared with its wild-type counterpart (Fig. 2C). The location of all of the reported *ESR1* missense germline mutations in prior studies [13–15] and somatic mutations in clinical samples from the Catalogue of Somatic Mutations in Cancer (COSMIC) database (https://cancer.sanger.ac.uk/cosmic); among these mutations, A207T is located in the DNA binding domain (DBD) (Fig. 2D).

### In silico prediction of ESR1 mutation

Three online bioinformatic programs, SIFT, PolyPhen-2 and MutationTaster, were applied to predict the potential pathogenicity of the *ESR1* mutation. The *ESR1* p.A207T mutation was predicted to be “damaging” and to affect protein function by SIFT, predicted to be “disease causing” by MutationTaster, and to be “probably damaging” with PolyPhen-2 (Table 2).

**Table 2:** In silico prediction of ESR1 mutation

Mutation	SIFT		Polyphen-2		Mutation Taster	
	Pathogenicity	Score(0–1)	Pathogenicity	Score(0–1)	Pathogenicity	PhastCons(0–1)
p.A207T	Damaging	0.000	Probably damaging	1.000	Disease causing	1.000

## Discussion

Prior studies revealed that ERα-knockout mice presented with clinical characteristics of PCOS, including disturbance of late maturation process of follicle development and failure of ovulation and the presence of large cysts, with few effects on development at the primordial, primary and antral follicle stages [9, 23]. Furthermore, ERα-knockout mice exhibited reduced ovulatory capacity, impaired mammary gland development and increased levels of serum estradiol and LH, compared with age-matched wild-type mice [23–26]. In addition, a prior study also revealed that two knock-in mutations in the AF-2 domain of *ESR1*, p.L543A and p.L544A, led to PCOS and elevated serum levels of estradiol and LH in mice [27]. To data, in humans, germline mutations in the *ESR1* gene have been reported in only three females [14, 15] and two males in human [13, 15]. All these patients exhibited clinical signs of estrogen insensitivity, namely, markedly elevated serum estrogen levels and mildly elevated gonadotropins (FSH and LH) [13–15]. Additionally, all *ESR1*-mutated women presented with bilateral multicystic ovaries and the absence of breast development [14, 15].

Here, we report a PCOS patient harboring a novel missense *ESR1* mutation, who had normal basal serum estradiol and LH levels and elevated FSH and AMH, levels and underwent a markedly prolonged ovarian stimulation period and poor response to this stimulus after pituitary downregulation with GnRH-a, exhibiting characteristics of estrogen insensitivity during the COH phase of an IVF cycle. In contrast, all previously reported females with *ESR1* germline mutations presented with markedly elevated levels of serum estrogen, FSH and LH, and complete estrogen insensitivity [14, 15]. Furthermore, breast development in this patient was unaffected, in contrast to the absence of breast development in previously described *ESR1*-mutated women [14, 15]. We speculate that the differences in clinical features and estrogen insensitivity severity between the present and prior studies [14, 15] could be due to the homozygous vs. heterozygous status of the *ESR1* mutation, as the wild type *ESR1* allele might confer some normal ERα function in this patient with heterozygous mutations.

The patient reported in the present study was diagnosed with PCOS and had multiple small follicles in the ovary, consistent with prior observations that *ESR1*-knockout and *ESR1*-mutated mice presented with characteristic features of PCOS [27]. The ovarian phenotypes of this patient seemed to be different from those observed previously, where bilateral enlarged multicystic ovaries, rather than PCOS phenotypes, were present [14, 15]. To date, all reported *ESR1*-mutated women, including those in the present and prior studies [14, 15], exhibited ovarian phenotypes; furthermore, PCOS is a multiple-factor complex disorder and a large proportion of women are diagnosed with PCOS at 30 years of age or older. Considering the young age of the previously reported female patients with *ESR1* mutations (18, 21 and 25 years old, respectively), it is uncertain whether these patients will develop PCOS in the future.

It will be interesting to see whether there is any difference among the female individuals harboring either homozygous or heterozygous ESR1 mutation. For the unaffected mother and sister of the proband harboring heterozygous *ESR1* mutation (p.R394H), they had normal puberty and underwent menarche at 14 and 15 years of age, respectively; furthermore, the proband’s mother has given birth to seven children and thus should be fertile [15], contrary to the secondary infertility of the patient in the present study. Notably, a newly published research reported two sisters from a consanguineous Jordanian family, displayed endocrine and ovarian defects of different severities despite they harbored the same homozygous ESR1 mutation (p.E385V); while we failed to get more information regarding the mutation status of ESR1 and the potential puberty and menarche condition for their mother [17]. For the homozygous ESR1 p.R157X men [13] and ESR1 p.Q375H women [14], we failed to know whether their mother and sister would exhibit similar phenotype with our sample [13]. Taken together, the clinical phenotype/symptoms of these females with either homozygous or heterozygous mutation in the *ESR1* gene might be affected by age, genetic and environmental factors, it might be more complex than we thought.

A limitation of the present study was that we failed to recruit the family members of *ESR1*-mutated patients to determine whether other females in the family also harbor *ESR1* mutation, and whether they might exhibit estrogen insensitivity, in certain circumstances. In addition, functional assays should be performed to clarify the potential mechanism of estrogen insensitivity conferred by the novel *ESR1* mutation.

In summary, we identified a novel heterozygous *ESR1* mutation in a Han Chinese PCOS patient who exhibited clinical signs of estrogen insensitivity. This study may provide new strategies for IVF therapy, especially for patients who exhibit estrogen insensitivity in the IVF cycle. Furthermore, WES is a cost-effective method to identify gene mutations that might be associated with clinically heterogeneous disorders and thus facilitate improvements in clinical therapy.

## Data Availability

The datasets used and/or analysed in the current study are available from the corresponding author on reasonable request.
